# Prehospital characteristics of COVID-19 patients transported by emergency medical service and the predictors of a prehospital sudden deterioration in Addis Ababa, Ethiopia

**DOI:** 10.1186/s12245-022-00463-z

**Published:** 2022-10-28

**Authors:** Lemlem Beza Demisse, Ararso Baru Olani, Micheal Alemayehu, Menbeu Sultan

**Affiliations:** 1 Department of Emergency Medicine, Addis Ababa University, Addis Ababa, Ethiopia; 2 College of Medicine and Health Science, Arbaminch University, Po. Box: 2021, Arbaminch, Ethiopia; 3 Tirunesh Beijing Hospital, Addis Ababa, Ethiopia; 4 Department of Emergency Medicine and Critical Care, St. Paul’s hospital millennium medical College, Addis Ababa, Ethiopia

**Keywords:** Prehospital, Sudden clinical deterioration, COVID-19, Addis Ababa, Ethiopia

## Abstract

**Background:**

Severally ill COVID-19 patients may require urgent transport to a specialized facility for advanced care. Prehospital transport is inherently risky; the patient’s health may deteriorate, and potentially fatal situations may arise. Hence, early detection of clinically worsening patients in a prehospital setting may enable selecting the best receiving facility, arranging for swift transportation, and providing the most accurate and timely therapies. The incidence and predictors of abrupt prehospital clinical deterioration among critically ill patients in Ethiopia are relatively limited.

**Study objectives:**

This study was conducted to determine the incidence of sudden clinical deterioration during prehospital transportation and its predictors.

**Methods:**

A prospective cohort study of 591 COVID-19 patients transported by a public EMS in Addis Ababa. For data entry, Epi data V4.2 and SPSS V 25 were used for analysis. To control the effect of confounders, the candidate variables for multivariable analysis were chosen using a p 0.25 inclusion threshold from the bivariate analysis. A statistically significant association was declared at adjusted relative risk (ARR) ≠ 1 with a 95 % confidence interval (CI) and a *p* value < 0.05 after adjusting for potential confounders.

**Results:**

The incidence of prehospital sudden clinical deterioration in this study was 10.8%. The independent predictors of prehospital sudden clinical deterioration were total prehospital time [ARR 1.03 (95%; CI 1.00–1.06)], queuing delays [ARR 1.03 (95%; CI 1.00–1.06)], initial prehospital respiratory rate [ARR 1.07 (95% CI 1.01–1.13)], and diabetic mellitus [ARR 1.06 (95%; CI 1.01–1.11)].

**Conclusion:**

In the current study, one in every ten COVID-19 patients experienced a clinical deterioration while an EMS provider was present. The factors that determined rapid deterioration were total prehospital time, queueing delays, the initial respiratory rate, and diabetes mellitus. Queueing delays should be managed in order to find a way to decrease overall prehospital time. According to this finding, more research on prehospital intervention and indicators of prehospital clinical deterioration in Ethiopia is warranted.

## Background

Prehospital care address a diverse set of diseases or conditions including infectious diseases, noncommunicable conditions, obstetrics, and injuries [1–3]. It comprises basic strategies with proven effectiveness, such as accessible and rapid transportation, and the deployment of personnel with at least basic life-support skills [1].

Nowadays, there is an increasing need for prehospital care both in high- and low-income countries as a consequence of the global increase in the emerging and evolving burden of diseases that need emergence medical services (EMS) [1, 3, 4]. In severe cases, COVID-19 may get worse and the illness may deteriorate to acute respiratory distress syndrome (ARDS) or multiple organ failure [5–8]. So, critically ill COVID-19 patients may require emergent transport to get advanced treatment such as non-invasive ventilatory support, invasive respiratory support, or admission to an intensive care unit [9–11]. However, the transportation of critically ill patients is not risk-free, and in some cases, the shortest transport could even be led to life-threatening conditions [12–15].

Several factors can contribute to sudden clinical deterioration in the prehospital setting and adverse events during transportation are one of the leading factors [16]. An increase in total prehospital time, unstable vital signs, neurological conditions, the profession of transferring personnel, and omission of necessary interventions were among the factors contributing to prehospital clinical deterioration [1, 17, 18]. Failure to detect and provide intervention for clinically deteriorating patients during emergency care also increases the risk of adverse events during the continuum of emergency care and may lead to devastating results [18–20]. Therefore, early identification of deteriorating patients is important in preventing or reducing the risk of sudden prehospital deterioration [11].

Early identification of clinically deteriorating patients in a prehospital setting may help to choose the appropriate receiving facility, rapid transportation, and provision of the most accurate and prompt interventions [21–23]. It could also help to anticipate the involvement of the senior staff such as the emergency department or critical-care professionals in the receiving facility [23]. Moreover, it has a proven impact on improving outcomes and management of critically ill patients [24].

Although early identifications of clinically deteriorating patients in prehospital settings have the aforementioned benefits, the available study in Ethiopia only focused on the clinical deterioration of hospitalized COVID-19 patients [25]. In addition, the available studies in Ethiopia that have assessed prehospital care independent of COVID-19 were mainly focused on the prevalence of prehospital care rather than addressing the incidence of prehospital clinical deterioration, types of prehospital care provided to the patients, and predictors of sudden deterioration [26–30]. Thus, there is a dearth of literature on the incidence of sudden clinical deterioration and its predictors in the prehospital setting in Ethiopia. So, this study aimed to examine the characteristics, incidence, and predictors of sudden prehospital deterioration among COVID-19 patients transported by EMS in Addis Ababa, Ethiopia.

## Methods

### Study design and setting

A prospective cohort study was implemented to assess the incidence of prehospital sudden deterioration among COVID-19 patients transported by public ambulances in Addis Ababa, Ethiopia. Addis Ababa is the capital city of Ethiopia and the seat of the African Union headquarter. In response to the COVID-19 outbreak in Ethiopia, the Addis Ababa city health bureau in collaboration with the Federal Ministry of Health established emergency operating centers (EOC) to provide prehospital care services to COVID-19 patients. The city has 10 dispatch centers and one central dispatch center dedicated to providing emergency medical services to COVID-19 patients. The centers have basic and advanced ambulances equipped with essential drugs and equipment. The ambulances were also staffed with different health care professionals including general practitioners and nurses. When an emergency call is received, the closest available ambulance is sent to the place to transport the COVID-19 patient to the nearest COVID-19 centers.

### Eligibility criteria

The study included all successful ambulance dispatches that transported COVID-19 patients aged 12 and up between May and August 2021. Failure to dispatch and insufficient information to determine sudden clinical deterioration during transportation to the receiving facility were exclusion criteria.

### Sample size and sampling procedure

The sample size for the study was calculated using single population proportion formula with the following consideration. The level of confidence (*α*) was set at 0.05 (*Z* (1-*α*) = 1.96), and the margin of error was considered at 0.05. It was reported that adverse events in the prehospital setting are one of the leading factors that cause sudden clinical deterioration in the prehospital setting [16]. Thus, the proportion of adverse events in the prehospital setting among COVID-19 patients in Addis Ababa was taken as 44.2% [31]. Considering 10% for contingency and a design effect of 1.5, the calculated sample size was 625. A cohort of COVID-19 patients who met eligibility criteria and were consecutively transported between May to August 2021 by Addis Ababa’s EOC was purposively recruited to the study.

### Data collection technique

The data collection tool was prepared by the investigators following reviews of previous works of literature [20, 32–34]. The data were collected by general practitioners and nurses working on the ambulance at each dispatch center in Addis Ababa. The tool consisted of information such as prehospital response time, source of EMS call, educational background of EMS providers, patients’ demographic data, clinical characteristics of the patients, and prehospital care provided to the patients.

### Outcome measures

The outcome of interest was sudden clinical deterioration during the prehospital transportation. The event must have occurred between the time of departure from the referring facility or home and the time of arrival at the receiving facility. Prehospital sudden clinical deterioration was measured by evaluating a change in any of the following physiological status components from the last recorded observations to the most recent [33]. These physiological changes include (A) change in pulse rate: either a sudden increase in pulse rate of 20 beats per minute above the previous reading, or the recently recorded pulse rate of greater than 110 beats per minute, or less than 50 beats per minute [33]; (B) change in blood pressure: either a sudden drop of blood pressure of 20 mmHg or more since the last reading or a fall below 90 mmHg systolic in the recent reading [33]; (C) change in respiratory rate: either a sudden increase in respiratory rate of 10 breaths per minute above previous reading or greater than 29 breaths per minute or less than 10 per breaths minute [33]; (D) change in the conscious state: either a sudden decrease in a conscious state of 2 points in either component (eye-opening, best verbal response, best motor response) of the Glasgow Coma Scale (GCS) or a newly recorded GCS score of <13 [33, 35]; and (E) cardio-pulmonary arrest [33].

### Data analysis

Epi data version 4.2 was used for data entry and SPSS version 25 was used for the analysis. Descriptive statistics such as frequency, percentage, mean, and standard deviation were used to summarize the findings, and tables and figures were used to present the information. A modified (Robust) Poisson regression was used to determine the relative risk summary metric and predictors of prehospital sudden clinical deterioration. We selected all candidate variables for multivariable analysis using a threshold for inclusion of *p* < 0.25 from bivariate analysis to control the effect of confounders. After adjusting for potential confounders, a statistically significant association was declared at adjusted relative risk (ARR) ≠ 1 with a 95% confidence interval (CI) and a *p* value less than 0.05.

### Operational definitions and definitions of terms

- Sudden prehospital deterioration: the patient was categorized as suddenly deteriorated if there was any change in the components of physiological parameters mentioned under the measurement section from the last recorded observations to the most recent.
- Response time: the time from the emergency call until arrival at the scene [36].
- On-scene time: the time from arrival at the scene until departure from the scene [36].
- Total prehospital time: the time from the emergency call until hospital arrival [36].
- Transport time: the time from scene departure until hospital arrival [36].
- Queueing delays: refers to delays when no ambulance is available to dispatch [37].

## Results

### Baseline characteristics

A total of 591 patients that met eligibility criteria were included in the analyses. The mean age of the study participants was 52.4 years with a standard deviation (SD) of 17.6 years. The majority of the COVID-19 patients included in this study were within the age group of ≥65 years followed by the age group of 45–54 years, which accounts for 173 (29.3%) and 111 (18.8%), respectively. Nearly two thirds of 388 (65.7%) study participants were male. Two out of three 394 (66.7%) COVID-19 patients included in this study were transported to the treatment center by ambulances staffed by general practitioners. The majority 404 (68.4%) of the COVID-19 patients were transported from the community to COVID-19 treatment centers (Table 1).

**Table 1:** Baseline characteristics of the COVID-19 patients transported by Addis Ababa’s emergency medical system, May to August 2021, Ethiopia

Variables	Frequency	Percentage
Age in years	(Mean=52.4; SD=17.6)	
<25	50	8.5
25–34	53	9.0
35–44	97	16.4
45–54	111	18.8
55–64	107	18.1
≥65	173	29.3
Sex		
Male	388	65.7
Female	203	34.3
Educational status of the patients		
Cannot read and write	45	7.6
Primary school	160	27.1
Secondary school	142	24.0
Diploma or vocational school	127	21.5
Bachelor degree and above	117	19.8
The profession of EMS provider		
Nurses	197	33.3
General practitioner	394	66.7
Source of the patient		
Community	404	68.4
Health care facility	187	31.6

### Clinical characteristics of the study participants

Of the total study participants, about 40% had a history of at least one chronic medical illness. Hypertension was the commonest 156 (26.4%) identified chronic illness among the study participants followed by diabetic mellitus 140 (23.7%). Among all patients transported by EMS provider, 121 (20.5%) had an initial prehospital systolic blood pressure of >130 mm Hg, 154 (26.1%) had an initial pulse rate of >100 beats per minute, the majority 356 (60.2%) had an initial prehospital respiratory rate of 21 to 30 per minute, nearly half 287 (47.7%) of the transported COVID-19 patients had an initial prehospital oxygen saturation of <90 percentage, while 26 (4.4%) of the patients had initial prehospital Glasgow coma scale (GCS) score of <13. Of the total transported patients, 64 (10.8%) of them experienced sudden prehospital clinical deterioration in the presence of an EMS provider. The incidence of sudden prehospital clinical deterioration identified in this study was 64 (10.8%) (Table 2).

**Table 2:** Clinical profiles of the COVID-19 patients transported by Addis Ababa’s emergency medical system, May to August 2021, Ethiopia

Variables	Frequency (n=591)	Percentage
Having at least one comorbid chronic illness		
Yes	234	39.6
No	357	60.4
Known diabetic mellitus		
Yes	140	23.7
No	451	76.3
Known hypertensive		
Yes	156	26.4
No	435	73.6
History of chronic heart disease		
Yes	35	5.9
No	556	94.1
Asthma or COPD		
Yes	50	8.5
No	541	91.5
Living with HIV/AIDS		
Yes	18	3.0
No	573	97.0
Initial prehospital systolic blood pressure in mmHg		
<90	8	1.4
90–130	462	78.2
>130	121	20.5
Initial prehospital pulse rate in beats per minute		
<60	6	1.0
60–100	431	72.9
>100	154	26.1
Initial prehospital respiratory rate, breaths per minute		
12–20	179	30.3
21–30	356	60.2
>30	55	9.3
Initial prehospital oxygen saturation in percentage		
<90	282	47.7
90–94	180	30.5
≥95	129	21.8
GCS		
<13	26	4.4
13–15	565	95.6
Prehospital sudden deterioration		
Yes	64	10.8
No	527	89.2

### Prehospital contextual characteristics

EMS performance times are shown in Table 3. The average response time in the present study was 46.2 min with an SD of 19.6 min. For about 30.1% of the COVID-19 patients, the response time was 31 to 45 min while for approximately one in five patients, 111 (18.8%), the EMS response time took over 60 min. The mean observed on-scene time in this study was 7.5 min. In the majority of 323 (54.7%) of the transported patients, the observed on-scene time was less than 5 min. The average total prehospital time spent transporting COVID-19 patients in this study was 81.4 min with an SD of 26.4 min. Prehospital queuing delays were observed in 82 (13.9%) COVID-19 patients transported by EMS. Delays in locating the patient address were experienced in 48 (8.1%) of the study participants (Table 3).

**Table 3:** Prehospital contextual characteristics of COVID-19 19 patients transported by Addis Ababa’s emergency medical system, May to August 2021, Ethiopia

Variables	Frequency	Percentage
Response time in minutes	Mean=46.2; SD=19.6	
≤15	19	3.2
16–30	136	23.0
31–45	178	30.1
46–60	147	24.9
>60	111	18.8
On-scene time in minutes	Mean=7.5; SD=4.7	
<5	323	54.7
6–10	179	30.3
11–15	61	10.3
>15	28	4.7
Total prehospital time in minutes	Mean=81.4; SD=26.4	
≤45	42 (7.1)	7.1
46–60	108	18.3
61–75	122	20.6
76–90	140	23.7
≥90	179	30.3
Queuing delays		
Yes	82	13.9
No	509	86.1
Delays in locating the patient address		
Yes	48	8.1
No	543	91.9
Omission of care due to lack of equipment		
Yes	152	25.7
No	439	74.3
Omission of care due to lack of medication		
Yes	80	13.5
No	511	86.5

### The prehospital interventions provided to the patients

**Fig. 1:**
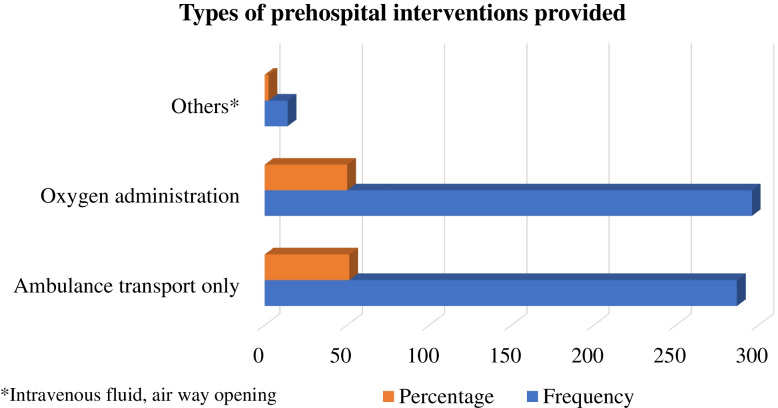
Types of prehospital intervention provided to COVID-19 patients

The majority of 296 (50.1%) of the patients received oxygen administration in prehospital. Meanwhile, slightly less than half of 287 (48.6%) of the patients received transportation by ambulance alone from an EMS provider (Fig. 1).

### Determinant of prehospital sudden deterioration

We have examined the influence of possible predictive factors on prehospital sudden clinical deterioration among COVID-19 patients. Total prehospital time independently determines prehospital sudden deterioration in this study [ARR 1.03 (95%; CI 1.00–1.06)]. Implying that the risk of prehospital sudden clinical deterioration was 1.03 times greater for those COVID-19 patients who had a total prehospital time of more than 60 min compared to those transported within 1 h. Similarly, queuing delays were associated with prehospital clinical deterioration in this study after controlling for the potential confounders [ARR 1.03 (95%; CI 1.00–1.06)]. COVID-19 patients who encountered queuing delays due to shortages of an ambulance to dispatch by dispatch centers were at about a 3% increase in the risk of sudden prehospital deterioration compared to those without queuing delays (Table 4).

**Table 4:** A modified Poisson regression analysis showing determinants of prehospital sudden clinical deterioration among the cohort of COVID-19 patients trasnsported by EMS, May to August 2021, Addis Ababa, Ethiopia

Variables	Prehospital sudden deterioration		CRR (95%) CI	ARR (95%) CI
	Yes	No		
The profession of EMS provider				
Nurses	33	164	1	
General practitioner	31	363	0.95 (0.92–0.98)	0.98 (0.96–1.01)
Response time in minutes				
≤30	9	146	1	
>30	55	381	1.04 (1.01–1.06)	1.02 (0.99–1.04)
Total prehospital time in minutes				
<60	10	139	1	1
≥60	54	338	1.03 (1.00–1.06)	1.03 (1.00–1.06)*
Queuing delays				
Yes	24	58	1.13 (1.06–1.19)	1.20 (1.04–1.16)**
No	40	469	1	1
Delays in locating the patient address				
Yes	11	37	1.07 (1.00–1.15)	1.01 (0.95–1.07)
No	53	490	1	1
The initial prehospital RR				
≤30 breath/minutes	52	483	1	1
>30 breath/minutes	12	43	1.07 (1.00–1.14)	1.07 (1.01–1.13)*
Having ≥1 comorbid chronic illness				
Yes	43	191	1.07 (1.04–1.10)	1.01 (0.96–1.04)
No	21	336	1	1
Known diabetic				
Yes	32	108	1.09 (1.05–1.13)	1.06 (1.01–1.11)*
No	32	419	1	1
Known hypertensive				
Yes	30	126	1.06 (1.03–1.10)	1.02 (0.98–1.07)
No	34	401	1	1
Chronic heart disease				
Yes	10	25	1.11 (1.02–1.21)	1.03 (0.95–1.12)
No	54	502	1	1
Asthmatic or COPD				
Yes	16	34	1.14 (1.05–1.23)	1.07 (0.98–1.15)
No	48	493	1	1
Omission of care due to lack of medication				
Yes	16	64	1.06 (1.01–1.11)	1.04 (0.99–1.09)
No	48	483	1	1
Omission of care due to lack of equipment				
Yes	27	125	1.05 (1.01–1.09)	1.01 (0.98–1.05)
No	37	402	1	1

*P < 0.05, **P <0.01, *1* reference, *CRR* crude relative risk, *ARR* adjusted relative risk, *CI* confidence interval

The initial prehospital respiratory rate was also independently determined prehospital clinical deterioration [ARR 1.07 (95%; CI 1.01–1.13)]. Implying that a COVID-19 patient with an initial respiratory rate of greater than 30 breaths per minute was about 7% times at increased risk of prehospital sudden clinical deterioration compared to their counterparts (Table 4). The study also found that COVID-19 patients with a history of diabetic mellitus were about 6% at higher risk of prehospital sudden clinical deterioration compared to those without a history of diabetic mellitus [ARR 1.06 (95%; CI 1.01–1.11)] (Table 4).

Although statistically significant associations were not observed on multivariate analysis, unadjusted analysis showed a significant association for the following variables: profession of EMS providers [CRR 0.95 (95%; CI 0.92–0.98)], EMS response time [CRR 1.04(95%;CI: 1.01-1.06)], delays in locating the patient address [CRR 1.07(95%; CI: 1.00-1.15)], having at least one comorbid illness [CRR 1.07(95%;CI:1.04-1.10)], hypertension [CRR 1.06 (95%; CI 1.03–1.10)], chronic heart disease [CRR 1.11 (95%; CI 1.02–1.21)], being asthmatic or COPD [CRR 1.14 (95%; CI 1.05–1.23)], omission of care due to lack of medication [CRR 1.06 (95%; CI 1.01–1.11)], and omission of care due to lack of equipment [CRR 1.05 (95%; CI 1.01–1.09)] (Table 4).

## Discussion

The present study showed that more than two thirds of the patients were transported from the community to receiving facility. Contrary to this finding, a previous study conducted in the same city reported that ambulance was mainly used for inter-facility transportation of critically ill patients. According to the study, 87.6% of ambulance-utilized patients were transported between health care facilities [38]. The disparities in the finding could be attributable to the fact that the present study was conducted among COVID-19 patients, and in response to the COVID-19 outbreak in Ethiopia, there were EMS services with organized dispatch centers, manpower, and ambulances that were dedicated to transporting COVID-19 patients from the community and between facilities as opposed to the previous study.

In severe cases, COVID-19 may lead to hypoxemic respiratory failure that may meet the criteria for acute ARDS [5–7]. Independent of COVID-19, ARDS has commonly been encountered in the prehospital setting and its management involves oxygen delivery and treatments of the underlying cause [39, 40]. In this study, oxygen administration was the commonest prehospital intervention provided to COVID-19 patients in addition to ambulance transportation. Oxygen administration using a facial mask and procedures such as intravenous line and fluid administration was rare. Advanced procedures such as non-invasive airway management and prehospital intubation were not considered although there was a need for such procedures. This could be due to the fact that the scope of prehospital practice in Ethiopia is not well established and there is also a lack of resources to provide advanced intervention in the prehospital setting. This finding suggests that there is a need to consider a two-tiered ambulance system for effective prehospital care with trained manpower on basic and advanced life support. However, the use of such a system should be done with caution as such systems need accurate classification of patient severity to avoid complications and under or over triage during ambulance dispatch [41].

Ambulance response time is a basic indicator of emergency medical services across the globe [42]. In the present study, the mean emergency response time was 46.2 min. Our findings were in line with the reported perceived ambulance waiting time by the residents of Addis Ababa city in the previous study, which reported an ambulance waiting time of 40 min [30]. However, the present findings were higher than the average response times reported from Brazil and Ghana, which reported 27 and 17 min, respectively [42, 43]. A possible explanation could be that the present study focused on the transportation of critically ill COVID-19 patients and ambulance that transport COVID-19 patients may need more extra time in preparation before the ambulance gets back into the service because of additional disinfectant protocols as compared to the ambulance that transport non-COVID-19 patients. The availability of ambulances to respond to emergency calls could be another reason for the discrepancy as nearly 14% of the patients in the present study experienced queuing delays due to a shortage of ambulances to dispatch. In addition, difficulty in locating the patient’s address and a notorious delay due to road traffic are of much concern in Addis Ababa. On the other hand, the mean prehospital time in this study was 81.4 min. The present findings were in line with the findings from Accra, Ghana, which reported an average prehospital transportation time of 82 min [43]. The present findings suggest that there is a need to reduce ambulance response time and total prehospital time to nationally and internationally accepted standards.

The incidence of prehospital sudden clinical deterioration in this study was 10.8%. Our figure is higher than some findings from Australia, which used similar predefined criteria to address the sudden prehospital clinical deterioration in trauma patients [32, 33]. The difference in the advancement of the EMS system between Australia and Ethiopia could be the reason for the disparity in the findings, as the EMS system in Ethiopia is a recent phenomenon [30, 44]. In addition, treatment-seeking delay by the patients or delayed inter-hospital transfer could be another factor as a significant proportion of the patients included in this study were critically ill and had unstable vital signs before transportations.

Our findings indicate that COVID-19 patients are at higher risk of sudden clinical deterioration in prehospital settings with increasing total prehospital time. A previous study conducted among trauma patients supported our findings [34]. We also identified that an increase in EMS response time was associated with an increased risk of sudden clinical deterioration in the prehospital setting without adjusting for potential confounders. Although we could not find literature on critically ill COVID-19 patients, it was reported that longer EMS response time was associated with prehospital adverse outcomes in trauma patients [43]. Moreover, queuing delays were another factor that independently determined prehospital sudden clinical deterioration in the present study. Further studies are needed to confirm the association between EMS response time, queuing delays, and prehospital sudden clinical deterioration in critically ill patients.

Respiratory distress in COVID-19 patients was reported as a considerable challenge for the prehospital EMS [45]. In the present study, we found an independent association between an increased initial respiratory rate and prehospital sudden clinical deterioration. In agreement with our findings, increased respiratory rate was independently associated with clinical deterioration among hospitalized COVID-19 patients [46, 47]. Previous findings also reported that COVID-19 patients may even rapidly deteriorate without showing any sign of respiratory distress or with little distress, which is called silent hypoxia [48, 49]. Therefore, identification of the initial respiratory rate could help in the early detection and prevention of prehospital sudden clinical deterioration in COVID-19 patients.

### Limitations

Despite the fact that this study used a prospective cohort design, the findings should be interpreted with vigilance. Variables such as transport distance, ambulance type, and COVID-19 severity level were excluded from the analysis because the majority of the data for these variables was missing. Future research incorporating the aforementioned variables may reveal a variety of findings. Besides that, this study only looked at prehospital outcomes. As a result, the current study did not investigate the relationship between prehospital characteristics and subsequent in-hospital outcomes. Further to that, the current study was conducted on COVID-19 patients and had geographical limitations because it only included public EMS in Addis Ababa. As a result, the study’s findings may be limited in their applicability to non-COVID-19 patients and other settings.

## Conclusion

Prehospital clinical sudden deterioration is relatively common among COVID-19 patients in Addis Ababa, with approximately one in ten patients experiencing sudden clinical deterioration in the presence of an EMS provider. Determinants of a sudden deterioration in the prehospital setting were total prehospital time, queuing delays, the initial respiratory rate, and diabetic mellitus. Most of the predictors of sudden prehospital clinical deterioration identified in this study are modifiable and could be averted. Implementing an action to reduce total prehospital time and queuing delays should be a management objective. In addition, early identification and management of COVID-19 patients with an increased initial respiratory rate could help in reducing the risk of sudden prehospital clinical deterioration. Moreover, this finding highlights the need for further research on prehospital care and predictors of prehospital clinical deterioration in Ethiopia.

## Data Availability

The datasets produced and/or analyzed during the current study are not publicly available even though public data sharing was not approved by the IRB, but they are available from the corresponding author (PI) upon reasonable request.
